# Evaluation of Infection Preventionists' Competencies Using the Association for Professionals in Infection Control and Epidemiology Competency Model in Tertiary Care Government Hospitals, Bahrain

**DOI:** 10.7759/cureus.65764

**Published:** 2024-07-30

**Authors:** Saleh F Sowar, Rommel Acunin, Tamer Abo Arisheh, Harold C Cabanalan, Safa Alkhawaja

**Affiliations:** 1 Infection Control, Government Hospitals, Manama, BHR; 2 Infection Prevention and Control, Government Hospitals, Manama, BHR; 3 Medicine, Salmaniya Medical Complex, Manama, BHR

**Keywords:** healthcare workers, infection preventionist, apic, competency, infection control

## Abstract

Introduction

Infection preventionists (IPs) are healthcare workers (HCWs) who received specialized training in infection prevention and control (IPC) to be able to deliver specific tasks to prevent and control infection transmission to patients, HCWs, and visitors in all healthcare settings. Most IPs started their professional life as nurses, physicians, microbiology, public health, or allied health specialties before moving to the IPC field, not necessarily with specialized training or diploma, which may need more focus on unified competency tool to evaluate their skills and strengths and improve IPC program outcome.

Aim

This study aimed to examine IPs' self-rated competency level using the Association for Professionals in Infection Control and Epidemiology (APIC) core competencies and to determine what factors influence the level of competency among IPs in Government Hospitals (GHs) in Manama, Bahrain.

Subject and methods

This cross-sectional study was conducted among non-certified IPs in Bahrain's GHs. A self-administered questionnaire was distributed among the targeted IPs using the Microsoft 365 form (Microsoft® Corp., Redmond, WA). The questionnaire includes socio-demographic characteristics (e.g., age, gender, years of experience, etc.), the APIC competency tool to assess IP competency, and the perceived effectiveness of the IPC program.

Results

Of the 17 IPs, 88.2% were females, and 47.1% were between 31 and 35 years old. Among the eight APIC core competencies, management and communication had the highest mean percentage score (84.2%), followed by preventing/controlling the transmission of infectious diseases (84.1%) and the identification of infectious disease processes (81.5%). The total mean competency score was 142.8 (SD: 20.3), with 70.6% classified as having a good level of competency. Increasing years of IP experience was the only factor associated with increased competency scores.

Conclusion

The level of IPC competency among "novice" or "becoming" proficient IPs was sufficient. Using the APIC competency tool, IPs showed competence in managing and communicating, preventing or controlling transmission of infectious agents, identifying infectious disease processes, and surveillance and epidemiologic investigations. Further research is needed to give more insights into the level of IPC competency of IPs in our region.

## Introduction

Infection preventionists (IPs) are healthcare workers who receive specialized training in infection prevention and control (IPC) to deliver specific tasks to prevent and control infection transmission to patients, healthcare workers (HCWs), and visitors in all healthcare settings. Most IPs started their professional life as nurses, physicians, public health, or allied health specialties before moving to the IPC field [1]. Internationally, there are wide differences in IPs' educational and professional development pathways, ranging from certification from various credentialing bodies such as the Certification in Infection Control (CIC) by the Certification Board of Infection Control and Epidemiology (CBIC) in the United States (US) to advance degrees, diploma, or certificates affiliated with universities in some countries such as Korea, Egypt, and Oman, while well-structured IPC training was limited or absent in some African countries [2,3]. The attention to IP competencies was highlighted more after the COVID-19 pandemic era [4]. Although the presence of IPs does not entirely eliminate the risk of hospital-associated infection (HAI) transmission, studies found a significant association between the presence of CIC-certified IP and lower multi-drug resistant organism (MDRO) infection rate, prevention outbreaks, and control infection transmission in healthcare facilities through the comprehensive implementation of evidence-based IPC practices [5-9]. Many renowned IPC organizations, such as the World Health Organization (WHO), Association for Professionals in Infection Control and Epidemiology (APIC), the Infection Prevention Society (IPS), and Infection Prevention and Control Canada (IPAC), developed a set of evidence-based core competencies for IPs in order to standardize the definition of the competent IPs and serve as a guide for IPs competency development and assessment [10-15]. Those core competencies were reviewed by many authors who identified similar competencies among those IPC bodies [16]. Based on competencies level, the APIC competency model categorized IP career stages as early stage (novice and becoming proficient), middle stage (proficient) if master the core competencies, for example, passing the CIC exam, and advanced stage (expert) if the IP serves as a role model and content expert in IPC [17]. Considering its importance, APIC recently released a new guide to facilitate IPs' advancement through the career stages. In addition to the IP core career stages outlined by the APIC competency model, this guide sheds light on two new stages in IP professional development, which are the IP paraprofessional stage (HCW who work under the direction of an IP) and IP leadership stages (IPs who lead teams and programs across an organization or system) and provided many resources and templates such as competencies self-assessment tools and professional development plan [18].

Literature review

Many published studies described the impact of IP training and competencies on the quality of IPC programs in healthcare facilities and the additional need for IPC expertise in other nontraditional healthcare settings. The APIC competency model was found to be effective in identifying the IP educational needs and supporting professional growth through the competencies self-assessment [19-21]. Bernard et al. [19] studied IPs' competency self-assessment to assess areas for improvement as a part of the professional development program for IPs in the US; their findings suggested a strong link between IPs years of experience and educational background and the reported self-assessed competency, the study recommended the APIC competency model as a useful tool to guide IPs professional development [19]. Similarly, a study was conducted in five specialized hospitals at Zagazig University in Egypt to evaluate the effectiveness of an APIC model-based training program; the study results showed improvement in the self-rated competency among the participants in pre and post the training program, which reflected the benefit of the self-assessment tool in closing gaps in IPs' knowledge and skills [22]. In another study in Japan in 2020, to identify competencies among Japanese certified nurses in IPC, findings showed a positive association between years of experience and self-rated level of competencies and helped the researchers identify competencies that need to be strengthened [23]. Data from the Eastern Mediterranean Region (EMR) showed variations in IPC educational opportunities, prerequisite qualifications, and the region's limited number of certified IPs; they highlighted the urgent need to support IPs' education and professional development [24-27]. To the best of our knowledge, there is no published data on the level of IPs' competencies in the Kingdom of Bahrain. Hence, this study was conducted to examine IPs' self-rated competency level for each of the eight APIC core competencies and to evaluate the relationship between independent variables and competency levels/certification status of IPs at Government Hospitals (GHs) in Bahrain.

## Materials and methods

A cross-sectional study was conducted on eligible "novices" or "becoming" proficient IPs working in GHs, Bahrain. Data were collected from May to June 2024. A self-administered questionnaire adopted from the APIC Novice and Becoming Proficient Self-Assessment Tool was utilized, and socio-demographics (i.e., age, gender, years in practice, etc.) were included. Participants were informed of the voluntary nature of this study and provided their informed consent before completing the questionnaire. A convenience sampling method was employed to recruit all "novice" and "becoming" proficient (non-CIC-certified) IPs in GHs. The total number of IPs in our institution was 25. The Research Committee for GHs of Bahrain approved this project (research approval serial no. 61-230524) on 23 May 2024.

Study area/setting

GHs are the main providers of secondary and tertiary government health services for all citizens and residents in Bahrain. It has an approximate capacity of 1,500 inpatient beds and comprises different health centers with various medical specialties, ranging from acute and emergency, psychiatry, rehabilitation, maternity, dialysis, long-stay, and outpatient primary/specialty care clinics [28]. The infection control group in GHs consists of 25 IPs with nursing backgrounds, one manager, and one infectious disease consultant physician as a director. Only the group director, manager, and two IPs are CIC-certified.

Measure

The APIC competency self-assessment tool, designed for "novice" or "becoming" proficient IP, which was initially developed in 2012 and updated in 2019, was adopted as a main data collection tool [29]. The tool was distributed using a webpage link through an online Microsoft 365 form (Microsoft® Corp., Redmond, WA). IP participants filled out the form through a self-assessed rating scale and comfort level according to their knowledge/skills/experience/confidence on each IPC practice area element using 4-point Likert scale categories (1 = No idea; 2 = Unsure; 3 = Some knowledge; 4 = Know it). The tool consists of eight core competency domains identified by CBIC and 44 items in IPC practice areas: identification of infectious disease processes (five items); surveillance and epidemiologic investigation (four items); preventing/controlling the transmission of infectious agents (17 items); management and communication (three items); education and research (two items); employee/occupational health (five items); environment of care (five items); and cleaning, sterilization, disinfection, and asepsis (three items). The reliability test of the APIC questionnaire, with ratings from good to excellent, has the following Cronbach alpha, respective to each domain: identification of infectious disease processes (0.778); surveillance and epidemiologic investigation (0.824); preventing/controlling the transmission of infectious agents (0.916); management and communication (0.908); education and research (0.679); employee/occupational health (0.905); environment of care (0.910); and cleaning, sterilization, disinfection, and asepsis (0.893). The overall reliability of APIC competency consisting of 44 items has a Cronbach alpha of 0.968 or 96.8%, suggesting an excellent internal consistency. Thus, the questionnaire is valid to use in this study.

All items, irrespective of each domain, were added to achieve the total scores. The overall competency score has been calculated by adding all 44 items. A score ranging from 44 to 176 was obtained. The higher the score, the higher the IP competency. Since no criteria were written for the APIC core competency score, we then applied 50% and 75% thresholds to classify competency scores into three levels. IP was considered as having poor competency if the total score was below 50%, 50%, and 75% were considered moderate, and above 75% was considered good competency [30].

Statistical analysis

The data were analyzed using the Statistical Product and Service Solutions(SPSS, version 26; IBM Corp., Armonk, NY). Descriptive statistics were given as numbers and percentages (%) for all categorical variables, while means and standard deviations were used to elaborate all continuous variables. The association between the total competency score and the socio-demographic characteristics of IPs was conducted using the independent sample t-test and the one-way ANOVA test. The normality test was performed using the Shapiro-Wilk test and the Kolmogorov-Smirnov test. According to the results, the competency score follows the normal distribution. Therefore, the parametric tests were applied. A p-value of less than 0.05 was considered statistically significant.

## Results

Seventeen non-certified IPs completed the survey (response rate: 68%). Table 1 presents the socio-demographic characteristics of the IPs. Specifically, 47.1% were between 31 and 35 years old, with females being dominant (88.2%). IPs with more than 10 years of experience in nursing bedside constitute 35.3%, while those with four to five years of experience as a full-time IP constitute 64.7%. Additionally, all IPs showed intention to take the CIC exam in the future, and 88.2% strongly agreed that attending courses in different hospitals is necessary for CIC exam preparation.

**Table 1:** Socio-demographic characteristics of non-certified IPs (n=17)

Study Data	N (%)
Age group	
25-30 years	03 (17.6%)
31-35 years	08 (47.1%)
36-40 years	03 (17.6%)
41-45 years	02 (11.8%)
>45 years	01 (05.9%)
Gender	
Male	02 (11.8%)
Female	15 (88.2%)
Years of nursing bedside experience	
<1 year	02 (11.8%)
1-3 years	01 (05.9%)
4-5 years	02 (11.8%)
6-10 years	06 (35.3%)
>10 years	06 (35.3%)
Years of experience as a full-time IP (as IP and/ or ICLN)	
<1 year	01 (05.9%)
1-3 years	04 (23.5%)
4-5 years	11 (64.7%)
6-10 years	0
>10 years	01 (05.9%)
Planned to take CIC in the future	
Yes	17 (100%)
No	0
Believed that attending courses outside the hospital is necessary for your CIC exam preparation?	
Strong disagree	0
Disagree	0
Neutral	0
Agree	02 (11.8%)
Strongly agree	15 (88.2%)

Regarding the assessment of the APIC competency tool (Table 2), the statement with the highest rating related to the identification of infectious disease processes domain was "Differentiate between colonization, infection, and contamination" (mean score: 3.35), while the least rating was the statement "Correlate clinical signs and symptoms with the infectious disease process" (mean score: 3.18). Regarding the surveillance and epidemiologic investigation domain, the statement with the highest rating was "Outbreak investigation" (mean score: 3.41), whereas "Interpretation of surveillance data" showed the lowest (mean score: 3.06). In terms of preventing/controlling the transmission of infectious agents domain, the top three statements with the highest ratings were "Appropriate selection, use, and disposal of personal protective equipment" (mean score: 3.82), followed by "Principles of safe injection practices" (mean score: 3.76) and "Patient placement, transfer, discharge" (mean score: 3.76), while ratings about the "antimicrobial stewardship" showed the least rating (mean score: 2.76). Regarding the management and communication domain, the highest rating statement was "communication and feedback (mean score: 3.53), while in the education and research domain, education showed the highest rating (mean score: 3.41). Pertaining to the environment of care domain, "Collaborate on the evaluation and monitoring of environmental cleaning and disinfection practices and technologies" showed the highest rating (mean score: 3.47), whereas "Recognize and monitor elements important for a safe care environment (e.g., Heating-ventilation-air conditioning, water standards, construction" was the lowest (mean score: 2.82). Finally, the statement with the highest rating for cleaning, sterilization, disinfection, and asepsis domain was "Identify and evaluate appropriate cleaning, sterilization, and disinfection practices" (mean score: 3.35).

**Table 2:** Assessment of IPs' competency using the APIC competency tool (n=17) Response has a category range from "no idea" coded with 1 to "Know it" coded with 4. APIC: Association for Professionals in Infection Control and Epidemiology

Competency item	Mean ± SD
Identification of infectious disease processes	
Differentiate between colonization, infection, and contamination	3.35 ± 0.70
Identify appropriate practices for specimen collection, transportation, handling, and storage	3.35 ± 0.49
Interpret the relevance of diagnostic and laboratory reports	3.24 ± 0.44
Differentiate between prophylactic, empiric and therapeutic uses of antimicrobials	3.18 ± 0.73
Correlate clinical signs and symptoms with infectious disease process	3.18 ± 0.64
Surveillance and epidemiologic investigation	
Outbreak investigation	3.41 ± 0.51
Collection and compilation of surveillance data	3.29 ± 0.59
Design of surveillance systems	3.18 ± 0.88
Interpretation of surveillance data	3.06 ± 0.97
Preventing/controlling the transmission of infectious agents	
Appropriate selection, use, and disposal of personal protective equipment	3.82 ± 0.39
Principles of safe injection practices	3.76 ± 0.56
Patient placement, transfer, discharge	3.76 ± 0.44
Identifying, implementing and evaluating elements of standard precautions/routine practices	3.76 ± 0.44
Identify and implement infection prevention and control strategies related to Hand hygiene	3.65 ± 0.49
Use of patient care products and medical equipment	3.65 ± 0.49
Transmission-based Precautions	3.59 ± 0.51
Cleaning, disinfection and sterilization	3.53 ± 0.51
Recall of potentially contaminated equipment, food, medications, and supplies	3.29 ± 0.77
Wherever healthcare is provided (e.g., patient care units, etc.)	3.24 ± 0.75
Influx of patients with communicable diseases	3.24 ± 0.75
Infection risks associated with medical procedures and devices (e.g., dialysis, endoscopy, etc.)	3.24 ± 0.66
Environmental pathogens (e.g., Legionella, Aspergillus)	3.12 ± 0.60
Collaborate with relevant groups in planning community/facility responses to biological disasters (e.g., anthrax, etc)	3.00 ± 0.79
Develop evidence-based/informed infection prevention and control policies and procedures	2.88 ± 0.98
Immunization programs for patients	2.88 ± 0.78
Antimicrobial stewardship	2.76 ± 0.75
Management and communication	
Communication and feedback	3.53 ± 0.62
Quality/performance improvement and patient safety	3.29 ± 0.77
Planning	3.24 ± 0.75
Education and research	
Education	3.41 ± 0.51
Research	2.82 ± 0.73
Employee/occupational health	
Assess the risk of occupational exposure to infectious diseases	3.35 ± 0.70
Collaborate with occupational health to recognize HCW who represent a transmission risk to others	3.18 ± 0.81
Collaborate regarding exposure management related to communicable diseases	3.00 ± 0.71
Collaborate with occupational health to evaluate IP-related data and provide recommendations	2.94 ± 0.75
Review and/or develop screening and immunization programs	2.71 ± 0.77
Environment of care	
Collaborate on the evaluation and monitoring of environmental cleaning and disinfection practices and technologies	3.47 ± 0.62
Collaborate with others to select and evaluate environmental disinfectant products	3.12 ± 0.86
Assess infection risks of design, construction, and renovation that impact patient care settings	3.06 ± 0.83
Provide recommendations to reduce the risk of infection as part of the design, construction, and renovation process.	3.06 ± 0.83
Recognize and monitor elements important for a safe care environment (e.g., HVAC, water standards, construction)	2.82 ± 0.88
Cleaning, sterilization, disinfection, and asepsis	
Identify and evaluate appropriate cleaning, sterilization, and disinfection practices	3.35 ± 0.86
Identify and evaluate critical steps of cleaning, high-level disinfection, and sterilization.	3.18 ± 0.73
Collaborate with others to assess products under evaluation for their ability to be reprocessed.	2.82 ± 1.07

Table 3 shows the descriptive statistics of APIC competency tools. It was revealed that the mean percentage score was higher in the management and communication domain (84.2%), followed by preventing/controlling the transmission of infectious agents domain (84.1%) and identification of infectious disease processes (81.5%), while employee/occupational health achieved the lowest ratings (76%). The overall mean competency score was 142.8 (SD: 20.3), with a mean percentage score of 81.1%. Accordingly, 70.6% were deemed to have a good level of competency, and 29.4% were moderate. None of them were considered to have poor competency levels.

**Table 3:** Descriptive statistics of the APIC competency tool score (n=17) APIC: Association for Professionals in Infection Control and Epidemiology

APIC Domains	No. of Items	Total Score	Accumulated Score, Mean ± SD	Mean Percentage Score
Management and communication	03	12	10.1 ± 1.98	84.2%
Preventing/controlling the transmission of infectious agents	17	51	57.2 ± 7.22	84.1%
Identification of infectious disease processes	05	20	16.3 ± 2.20	81.5%
Surveillance and epidemiologic investigation	04	16	12.9 ± 2.46	80.6%
Education and research	02	08	6.24 ± 1.09	78.0%
Cleaning, sterilization, disinfection, and asepsis	03	12	9.35 ± 2.45	77.9%
Environment of care	05	20	15.5 ± 3.47	77.5%
Employee/occupational health	05	20	15.2 ± 3.19	76.0%
Total APIC competency score	44	176	142.8 ± 20.3	81.1%
Level of competency	N (%)			
Poor	0			
Moderate	05 (29.4%)			
Good	12 (70.6%)			

**Figure 1:**
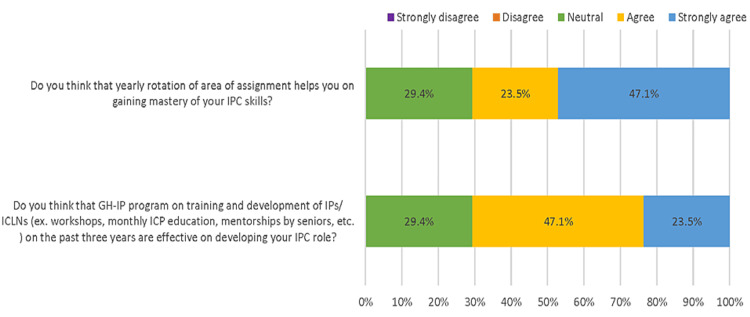
Effectiveness of the GH-IP development program

Figure 1 illustrates the perceived effectiveness of the GH-IP development program. It can be observed that 47.1% strongly agreed that yearly rotation of area of assignment could help in gaining mastery of IPC skills, and 23.5% strongly agreed that the GH-IP program on training and development of IPs over the past three years has been effective in developing IPC role.

**Figure 2:**
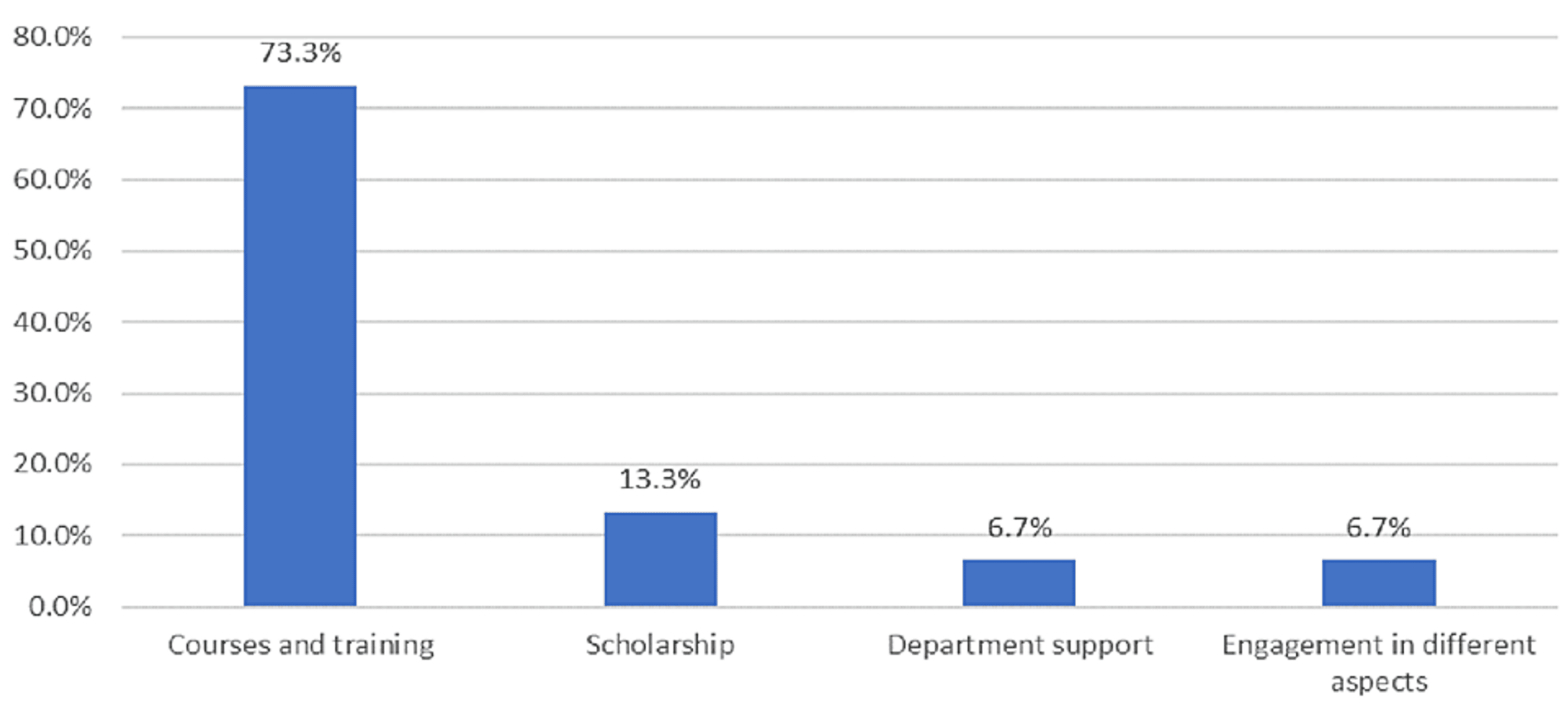
Perceived method to improve the GH-IP development program

Figure 2 depicted that the most prominent method to improve the GH-IP development program was courses and training (73.3%), followed by scholarship (13.3%).

Measuring the association between the total competency score and the socio-demographic characteristics of the IPs found that an increasing competency score was more associated with increasing years of IP experience (T=-2.735; p=0.015). No significant differences were observed between the competency scores in relation to age, gender, and years of nursing bedside experience (p>0.05) (Table 4).

**Table 4:** Association between total competency score and the socio-demographic characteristics of non-certified IPs (n=17) ^a^P-value was calculated using an independent sample t-test; ^b^P-value was calculated using the one-way ANOVA test; ^**^Significant at p<0.05 level.

Factor	Competency Score (176), Mean ± SD	T/F-test	P-value
Age group^a^			
≤35 years	142.2 ± 15.8	-0.156	0.878
>35 years	143.8 ± 29.5		
Gender^a^			
Male	144.0 ± 5.66	0.089	0.930
Female	142.6 ± 21.6		
Years of nursing bedside experience^b^			
≤5 years	155.3 ± 11.8	0.424	0.664
6-10 years	144.3 ± 15.6		
>10 years	142.3 ± 26.7		
Years of experience as a full-time IP (as IP and/ or ICLN) ^a^			
≤3 years	125.2 ± 15.1	-2.735	0.015 **
>3 years	150.1 ± 17.8		

## Discussion

This study examined the level of IP competency among infection control preventionists in GHs in Bahrain. To our knowledge, this is the first study conducted in Bahrain that discussed IPs' competency toward infection and prevention control, which could be an important contribution to the literature given the challenges HCWs face in controlling HAIs. Hence, the findings of this study could provide insights into the competency levels of "novice" or "becoming" proficient IPs toward IPC.

Level of competency

Using the APIC competency tool, the overall competency level of IPs on IPC was high (94.1%). The overall mean APIC competency score was 142.8 out of 176 points, with a mean percentage score of 81.1%. We noticed that this is the first study in Bahrain that managed to calculate the overall competency levels of IPs toward IPC. Hence, more investigations are required to confirm this finding. Furthermore, based on the given criteria, 12 out of 17 IPs (70.6%) were regarded as having good competencies. The effectiveness of the IPC program cannot be achieved without having knowledgeable and competent IPs. Our results suggest that IPs in GHs, Bahrain, are equipped with sufficient knowledge to be called competent in the IPC program. This is crucial in optimizing IPC programs in a healthcare facility since a competent IP could guide staff orientation, improve HAI preventive measures, improve patient outcomes, and promote systematically advanced practice in IPC.

Assessment of APIC competency core domains

Among the eight core APIC competencies, management and communication came atop, followed by preventing and controlling infection transmission, identifying infectious disease processes, and surveillance and epidemiologic investigations, while the employee or occupational health, research involvement, environment care, cleaning, sterilization, disinfection, and asepsis necessitates more improvement. In Egypt [22], IPs were experts and proficient in preventing/controlling the transmission of infectious agents but novices in surveillance and epidemiologic investigations, as well as employee/occupational health, while approaching proficiency in management and communication. However, among Japanese IPs [23], their competencies were high in the prevention and control of transmission of infectious agents, as well as education and research, whereas employee/occupational health was low. However, the score of each core competency did not reach at least 70%. The author suggested that the competencies of IPs need to be strengthened as necessary for career growth. Notwithstanding these reports, Tannous et al. [25] reported that IPC members in the Middle East and North Africa (MENA) found that surveillance was the most challenging task, followed by isolation and investigation of the outbreak. In their scenario, countries in MENA achieved satisfactory ratings in terms of IPC programs, but staffing needs improvement may vary significantly between each country associated with resources.

Significant factor of competency

Data from our study indicate that years of experience in IPC practice was the only factor of increased competency, suggesting that IPs who had more years of practice demonstrated higher competency scores than the IPs who had less experience (p=0.015). However, the competency score did not vary significantly by age, gender, and years of nursing bedside experience (p>0.05). This is in agreement with the study of Bernard et al. [19]. The study suggests that IPs with fewer years of experience were likelier to have lower competency levels [19]. In support of these accounts, Misao et al. [23] found that increasing career levels were correlated with increasing competency scores.

GH-IP development program

Our institutional policies related to the GH-IP development program selected IPs based on recommendations and interviews. Once recruited, a full month of intensive orientation to the new IP is given. They will be assigned an experienced IP counterpart who will provide frequent feedback and close supervision of their day-to-day induction process. Then, they will be assigned to specific locations and tasks with annual rotation, followed by monthly focus-grouped educational activities. However, our results suggest that only 47.1% strongly agreed that the yearly area of assignment rotation is beneficial in IPC skills, and only 23.5% strongly agreed that GH-IP development programs are effective in career development as an IP. CIC certification is important because it can validate competency levels within the profession. According to the report done by Hsu et al. [8], CIC-certified IPs may have a more robust understanding than other HCWs toward indications for certain infection prevention practices and were likelier to suggest the implementation at the current hospital settings, particularly when the head IP has certification [8]. However, a study done in Saudi Arabia highlighted deficiencies with the IPC program. Saudi Arabian IPC systems may be unable to act rapidly on new information due to the fact there is inadequate standardization across the fundamentals of infection control. Appropriate action to upgrade such systems becomes a challenge. The author, therefore, suggested that healthcare settings in Saudi Arabia must evaluate the need to develop, present, and assess an active, effective IPC program that connects the mandate and goal of diminishing the risk of HAIs and enhancing healthcare safety [27].

Future plan for CIC certification

All IPs who participated in this study showed their intention to obtain CIC certification in the future and nearly all (88.2%) strongly agreed that participation in different IPC-related courses is an important preparation for the CIC examination. Further, IPs perceived courses and training as the most prominent method to improve the GH-IP development program; other methods, such as scholarship, departmental support, and engagement in different aspects of a development program, were less prominent. Corroborating these reports, Kalp et al. [20] found that half of the IPs indicated that their competency levels in the classification of infectious disease process were mainly obtained from professional development through courses and training, followed by formal education, current practice, and self-learning [20]. The value of certification in IPC is significant in promoting patient care, safety, regulatory compliance, certification process and standards, professionalism, competency, and career growth [5].

Limitations

Some limitations may limit the generalizability of this study. First, a small sample size (N=17) limited the findings' generalizability and made applying the results to a bigger population challenging. Second, only two males were IPs; hence, the gender comparison is inconclusive. Third, being a cross-sectional study requires a bigger sample size to represent the study population accurately; however, the small size limits the generalizability of the study outcome.

Recommendations

Based on our findings, we recommend utilizing the existing IP competency self-assessment models, such as those from APIC, which are powerful tools for establishing an IP development program. This will allow IPC leadership to identify priorities for the training needs of IP staff. IP competency self-assessment can be done annually by IPs to plan their individual development; more support for developing the GH-IP training program and for scholarships is needed to expand the IPs' knowledge and clinical experience. Finally, a replication of this study discipline, probably a national study, is recommended, targeting all IPs nationwide. This will give us a better view of IPs' competency toward IPC in Bahrain's public healthcare settings.

## Conclusions

There was adequate IPC competency among IPs working at GHs in Bahrain. Increasing years of experience were associated with increasing levels of competencies. IPs were highly competent in management and communication, infection transmission prevention, identification of infectious disease processes, and surveillance and epidemiologic investigations. Further, IPs believe courses and training are keys to improving the GH-IP development program in our region. All participants reported that they are planning to take CIC certification.
